# Prosthetic graft replacement of a large subclavian aneurysm in a child with Loeys–Dietz syndrome: a case report

**DOI:** 10.1093/ehjcr/ytaa163

**Published:** 2020-08-23

**Authors:** Mohamed Sobh, Inga Voges, Tim Attmann, Jens Scheewe

**Affiliations:** Department of Congenital Heart Disease and Paediatric Cardiology, University Hospital Schleswig-Holstein, Arnold-Heller-Str. 3, Kiel, Germany; Department of Congenital Heart Disease and Paediatric Cardiology, University Hospital Schleswig-Holstein, Arnold-Heller-Str. 3, Kiel, Germany; Department of Cardiovascular Surgery, University Hospital Schleswig-Holstein, Arnold-Heller-Str. 3, 24105 Kiel, Germany; Department of Cardiovascular Surgery, University Hospital Schleswig-Holstein, Arnold-Heller-Str. 3, 24105 Kiel, Germany

**Keywords:** Loeys–Dietz syndrome, Cardiovascular magnetic resonance imaging, Subclavian artery aneurysm, Case report

## Abstract

**Background:**

Loeys–Dietz syndrome (LDS) is a genetic connective tissue disorder, which is characterized by rapid development of aortic and peripheral arterial aneurysms. Loeys–Dietz syndrome has some overlapping phenotypic features with other inherited aortopathies such as Marfan syndrome. However, LDS has a more aggressive vascular course with patient morbidity and mortality occurring at an early age.

**Case summary:**

We present the rare case of an 11-year-old girl with LDS who underwent valve sparing aortic root replacement at the age of 2.9 years with good results. She had routine follow-up cardiovascular magnetic resonance imaging and was found to have a large aneurysm of the right subclavian artery. After multidisciplinary team discussion, successful surgical resection with prosthetic graft replacement of the right subclavian artery was performed.

**Discussion:**

This case illustrates that large aneurysms of aortic branches can already develop in childhood and underlines the need for frequent follow-ups including cross-sectional imaging and multidisciplinary team management.

**Learning points**

- Children with Loeys–Dietz syndrome (LDS) are at risk for development of large vascular aneurysms.
- For optimal management, a multidisciplinary team approach and frequent cross-sectional imaging studies in LDS patients seems to be mandatory.

## Introduction

Loeys–Dietz syndrome (LDS) is an autosomal dominant disease affecting the connective tissue.^1^ It is caused by mutations in the genes encoding transforming growth factor β receptors 1 and 2 (TGFBR1 and TGFBR2, respectively).^1^^,^^2^ Typical features include hypertelorism, a bifid uvula or cleft palate, and generalized arterial tortuosity with widespread vascular aneurysms and dissections.^2^ Although, LDS and inherited aortopathies such as Marfan syndrome have overlapping phenotypic features, LDS is characterized by a more aggressive vascular course with complications developing at smaller aortic dimensions.^3^ In addition, patients with LDS present with more diffuse arterial disease involving also the iliac, mesenteric, and intracranial arteries.^3^ Loeys–Dietz syndrome patients were found to be at risk for aneurysm rupture already in childhood.^4^

## Timeline

**Table:** 

At 13 months of age	Interventional closure of a patent ductus arteriosus
At 2.9 years of age	valve-sparing aortic root replacement
At 3 years of age	Sternum revision, debridement
At 4 years of age	Cardiovascular magnetic resonance imaging (MRI)
At 6 years of age	Cardiovascular MRI
At 11 years of age	Cardiovascular MRI and MRI of the intracranial vessels
At 11.9 years of age	Prosthetic replacement of the right subclavian artery

## Case presentation

Here, we present an 11-year-old patient with LDS. This girl developed an early and rapid progressive enlargement of the aortic root (maximum aortic root size 30 mm, *z*-score > 7) and therefore underwent valve-sparing aortic root replacement at the age of 2.9 years using a 22 mm Albograft (AlboGraft^®^ polyester vascular graft, LeMaitre Vascular GmbH, Sulzbach/Ts., Germany).

Follow-up cardiovascular magnetic resonance imaging (CMR) under sedation in 2010, 2011, and 2013 showed good operative results with no evidence of dissection or aneurysm of the aorta. She was lost to follow-up for 5 years from our centre but was then referred for another CMR scan.

**Figure 1:**
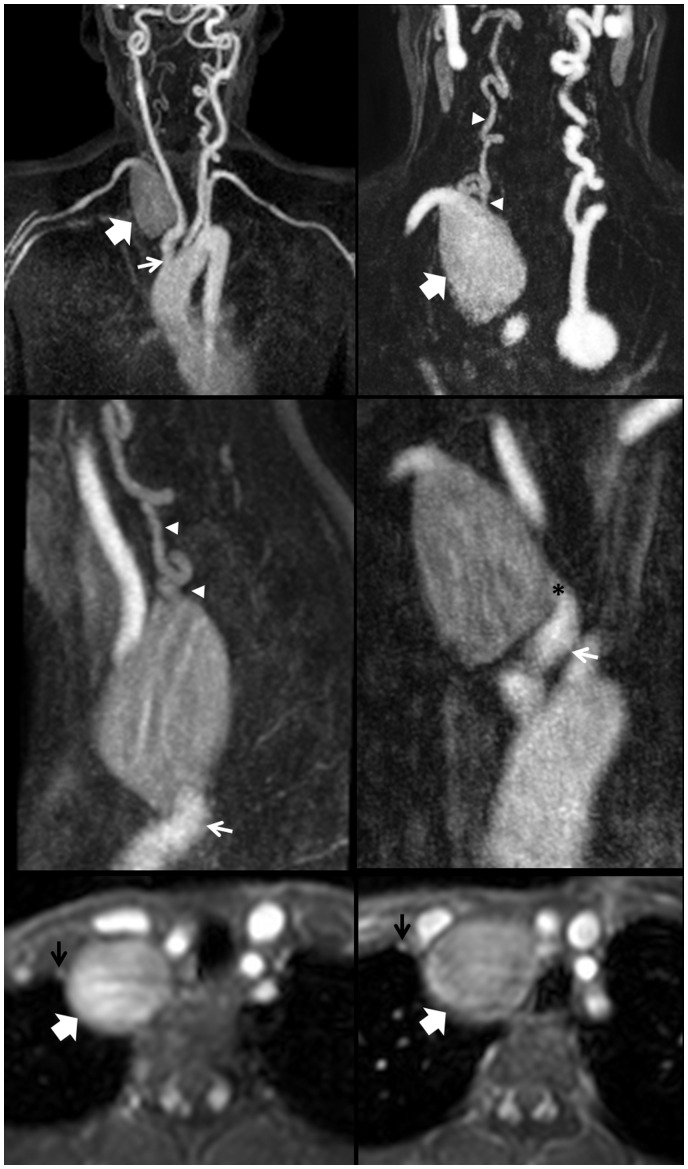
Contrast-enhanced magnetic resonance imaging with maximum intensity projection and multiplanar reconstruction showing large right subclavian artery aneurysm (large white arrows) and its entrance (black asterisk). The origin of the right vertebral artery from the aneurysm (white arrowheads) as well as the relationship to the brachiocephalic artery (small white arrows) and the right internal mammary artery (black arrows) is also demonstrated.

**Figure 2:**
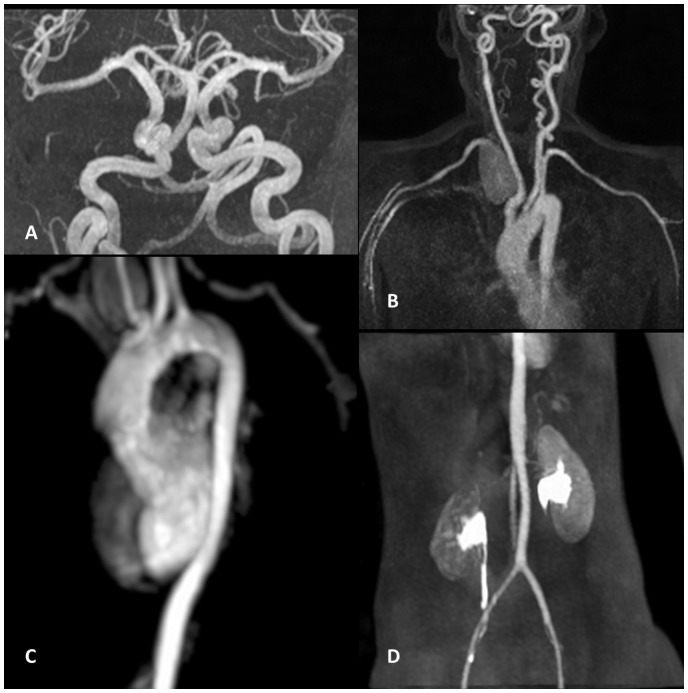
Maximum intensity projection from contrast-enhanced magnetic resonance angiography demonstrating the brain and head and neck vessels (*A* and *B*), the thoracic and abdominal aorta (*C* and *D*) as well as the iliac and femoral arteries.

On examination, we saw an 11-year-old girl in good general condition. Auscultation revealed a continuous murmur in the suprasternal notch area and at the right side of her neck. In addition, there was a soft diastolic murmur at the left parasternal border. Her chest was clear. Medication at that time consisted of metoprolol and captopril. Cardiovascular magnetic resonance imaging showed a large aneurysm (∼50 × 30 mm) at the origin of the subclavian artery (*Figure 1*). Additional magnetic resonance imaging (MRI) of the head and neck vessels as well as the abdominal aorta and iliac and femoral arteries demonstrated considerably elongated neck vessels as well as generalized elongation and slight ectasia of the basal brain vessels (*Figures 1* and *2*), no other aneurysms were detected. The right vertebral artery was arising from the large subclavian aneurysm (*Figure 1*). Magnetic resonance imaging of the head and neck vessels gave the impression that it was less well perfused possibly contributing only very little to the basilar artery flow.

The ascending aorta distal to the Albograft prosthesis had a diameter of 27 × 34 mm. The aortic valve showed mild regurgitation (regurgitant fraction 16%).

After a multidisciplinary discussion of the findings with the colleagues of interventional radiology and cardiac and vascular surgery, the indication for surgical resection with prosthetic replacement of the right subclavian aneurysm was made.

Surgical removal of the aneurysm and prosthetic replacement of the right subclavian from the brachiocephalic trunk to the exit of the right mammary artery with a 7 mm Gore-Tex-Vascular Graft (GORE-TEX^®^, W. L. Gore & Associates, Inc., AZ, USA) was performed via median sternotomy with the use of heart lung machine on the beating heart in moderate hypothermia (26°C) without any complications. Apart from the experience at our centre, the reason for median sternotomy was that the patient needed revision of the sternum due to a severe form of pectus carinatum. Cooling was performed for cerebral protection because it was not clear at the beginning of cardiopulmonary bypass if the right carotid artery had to be clamped during the procedure. The MRI finding of a small/hypoplastic right vertebral artery was confirmed during the operation, and therefore, the vessel was transected.

**Figure 3:**
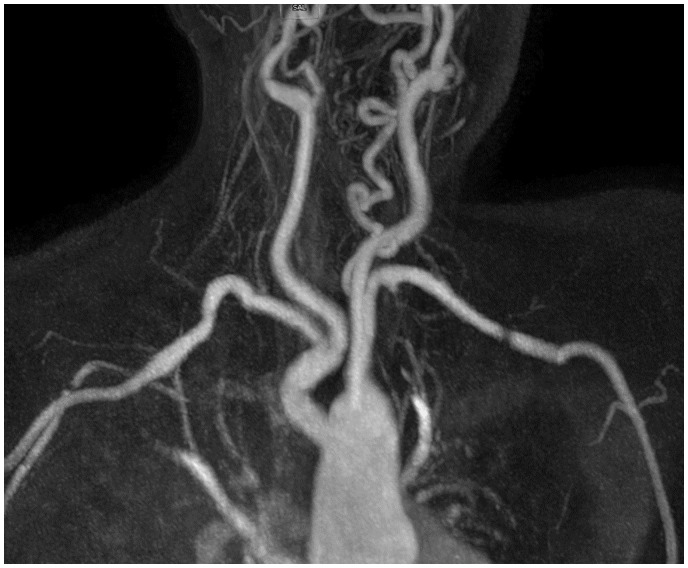
Maximum intensity projection from contrast-enhanced magnetic resonance angiography showing right subclavian artery after removal of the aneurysm and prosthetic graft replacement.

A follow-up CMR scan 1 month after surgery demonstrated a patent and non-obstructive Gore-Tex-Vascular graft which was used for the prosthetic replacement of the subclavian artery (*Figure 3*). On her most recent outpatient assessment, the patient was asymptomatic.

## Discussion

We demonstrate the rare finding of a large right subclavian aneurysm in an 11-year-old girl with LDS. The patient underwent successful surgical resection and prosthetic graft replacement of the aneurysm after multidisciplinary discussion.

Loeys–Dietz syndrome represents a potentially life-threatening cardiovascular disease with poor prognosis because of its sudden early onset (usually acute dissection) in previously undiagnosed patients.^5^ A study by Williams *et al*.^6^ showed aggressive aortic aneurysmal disease in LDS patients with a propensity towards rupture and dissection at a young age (mean age 9.2 ± 5.7 years) and with smaller aortic diameters than in other connective tissue disorders, particularly in the ascending aorta. That vascular complications in LDS patients can occur already at an early age was also demonstrated by Malhotra *et al*.^4^ They presented a 3 months old boy with LDS who had aortic dissection of the descending thoracic aorta. Aneurysms of the subclavian artery have been reported only in few adult cases.^7^^,^^8^

In our patient, results after surgical removal and graft replacement of the right subclavian artery were excellent. However, due to the aggressive nature of LDS and reported high reintervention rates,^9^ frequent follow-up examinations including yearly magnetic resonance imaging studies from head to pelvis^6^ for early detection of vascular complications are necessary.

## Conclusion

Our case demonstrates that LDS patients can develop large aneurysm of the head and neck vessels already during childhood. Life-long and frequent follow-up examinations of the cardiovascular status within a multidisciplinary team and including cross-sectional imaging seem mandatory.

## Lead author biography

**Figure:**
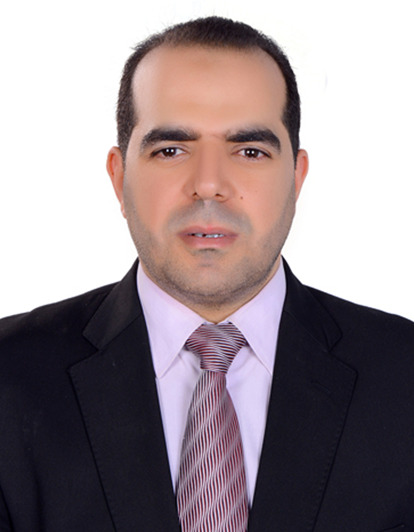


Dr Mohamed Sobh is a fellow in congenital cardiovascular magnetic resonance imaging (MRI) in the Department of Congenital Heart Disease and Paediatric Cardiology at the University Hospital Schleswig-Holstein, Campus Kiel. He has finished his training in paediatrics and paediatric cardiology in Egypt at the Cairo University. Currently, he focuses on clinical cardiovascular MRI imaging as well as on a research MRI project about the right ventricular function in patients with hypoplastic left heart syndrome.

## Supplementary material

Supplementary material is available at *European Heart Journal - Case Reports* online.

**Slide sets:** A fully edited slide set detailing this case and suitable for local presentation is available online as Supplementary data.

**Consent:** The authors confirm that written consent for submission and publication of this case report including images and associated text has been obtained from the patient in line with COPE guidance.

**Conflict of interest:** none declared.

## Supplementary Material

ytaa163_Supplementary_Data
