# Supplementary material for: Prosthetic graft replacement of a large subclavian aneurysm in a child with Loeys–Dietz syndrome: a case report
Source: Eur Heart J Case Rep. 2020 Aug 23;4(5):1–4. doi: 10.1093/ehjcr/ytaa163 (PMC7649448; doi:10.1093/ehjcr/ytaa163)
Supplement: ytaa163_Supplementary_Data [file ytaa163_supplementary_data.zip › ytaa163_Supplementary_Data/Slides iv_01052020.pptx]

## Slide 1
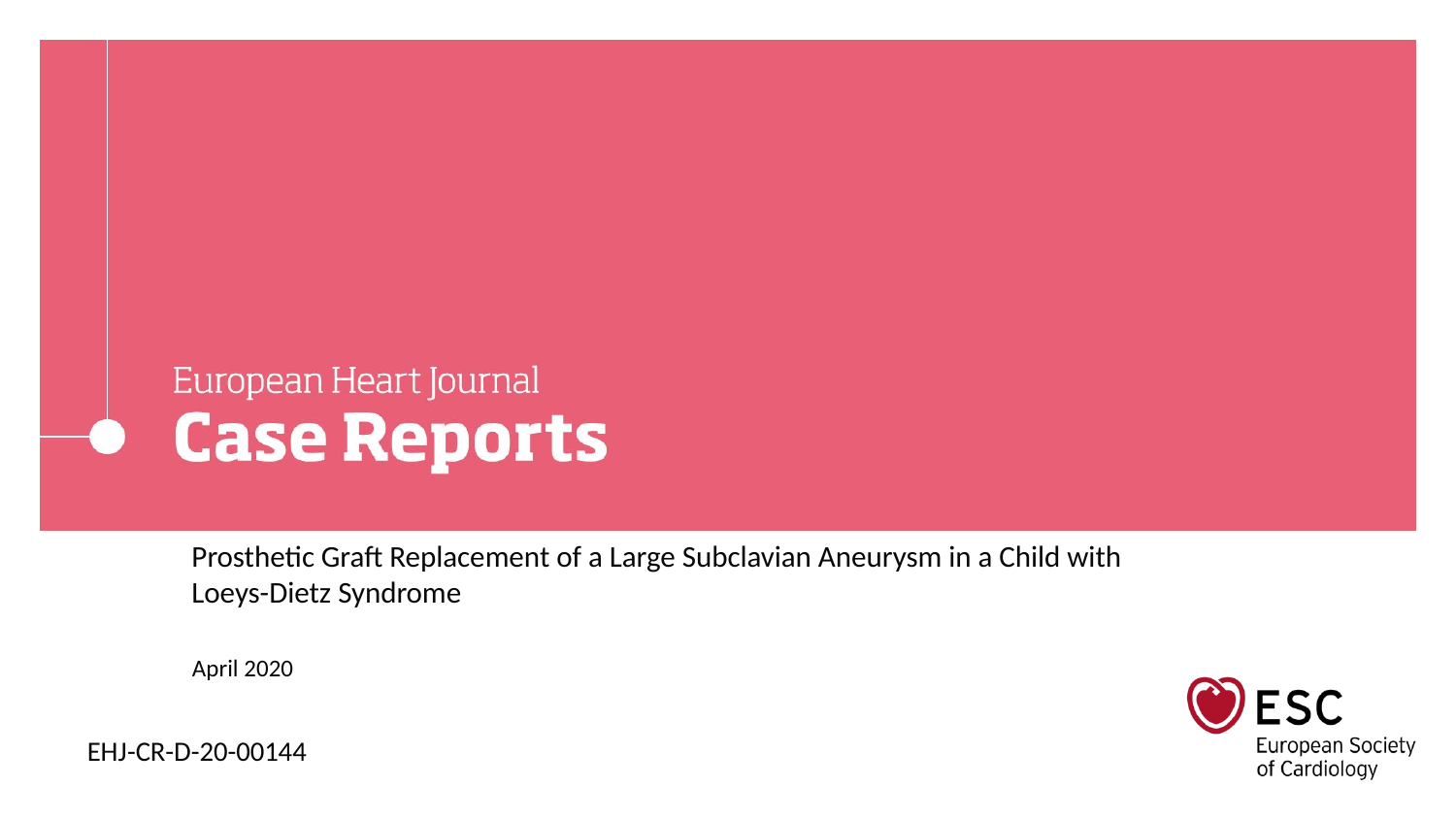

# Prosthetic Graft Replacement of a Large Subclavian Aneurysm in a Child with Loeys-Dietz Syndrome
April 2020
 EHJ-CR-D-20-00144

## Slide 2
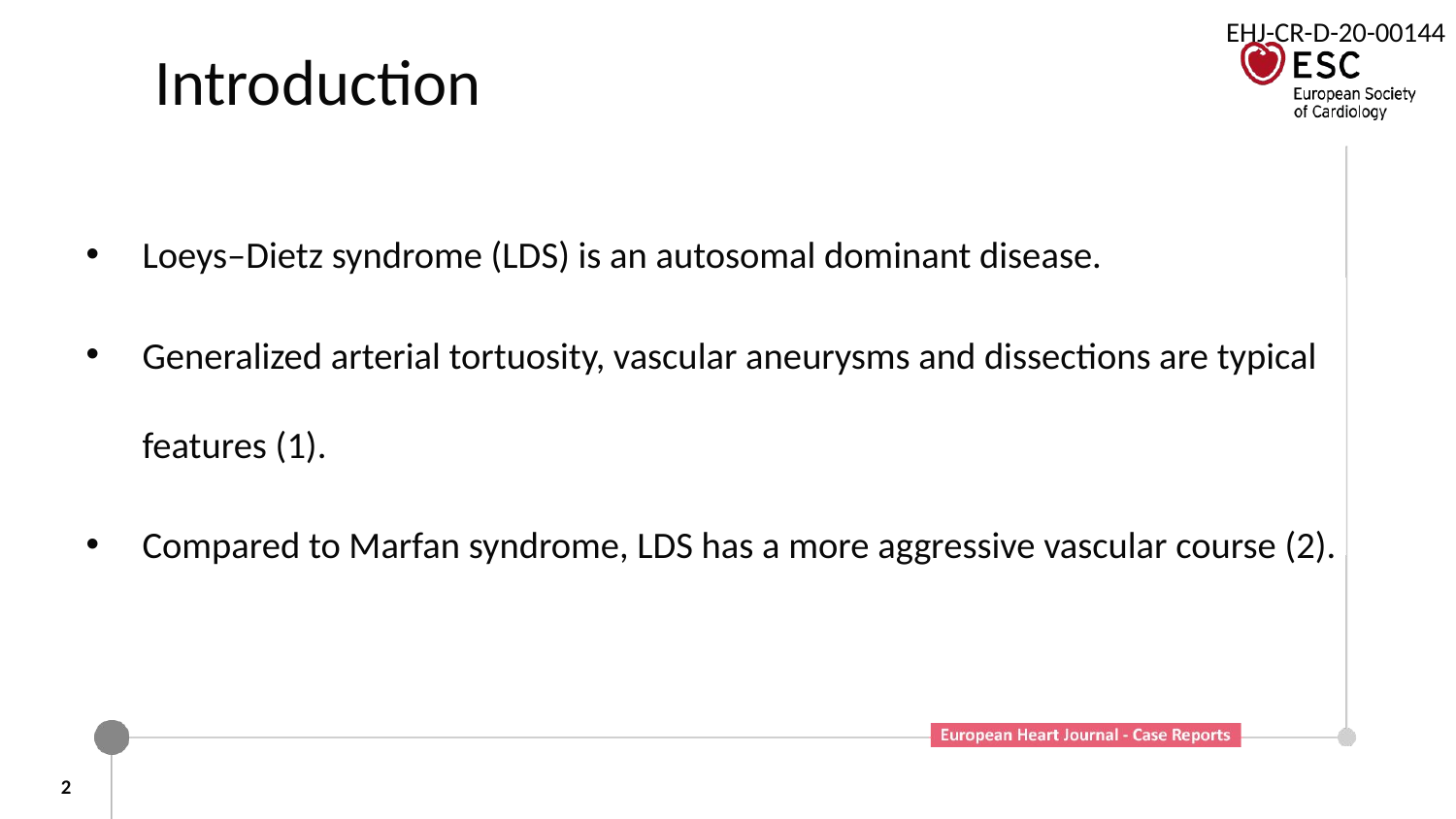

EHJ-CR-D-20-00144
# Introduction
Loeys–Dietz syndrome (LDS) is an autosomal dominant disease.
Generalized arterial tortuosity, vascular aneurysms and dissections are typical features (1).
Compared to Marfan syndrome, LDS has a more aggressive vascular course (2).
2

## Slide 3
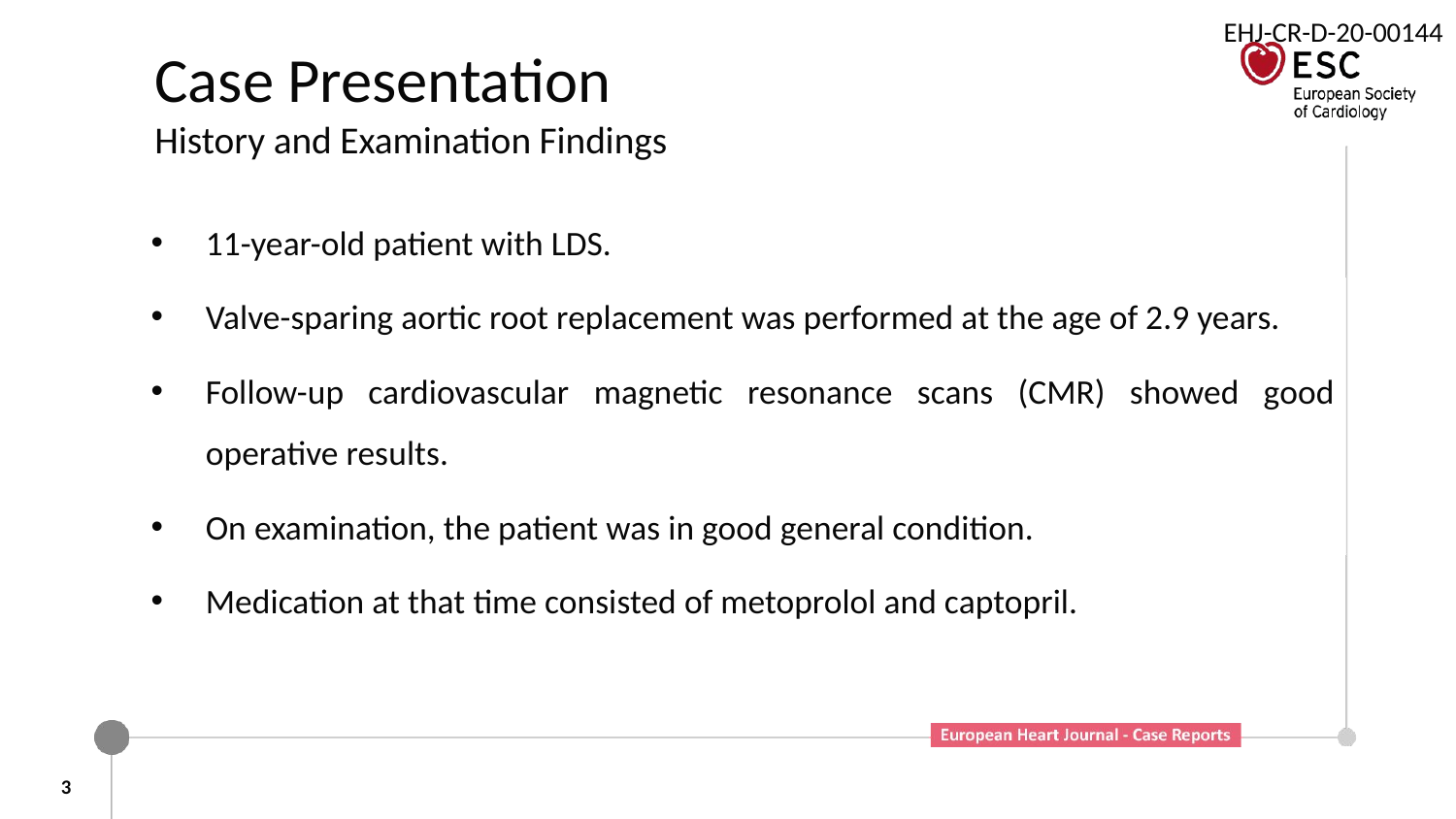

EHJ-CR-D-20-00144
# Case Presentation History and Examination Findings
11-year-old patient with LDS.
Valve-sparing aortic root replacement was performed at the age of 2.9 years.
Follow-up cardiovascular magnetic resonance scans (CMR) showed good operative results.
On examination, the patient was in good general condition.
Medication at that time consisted of metoprolol and captopril.
3

## Slide 4
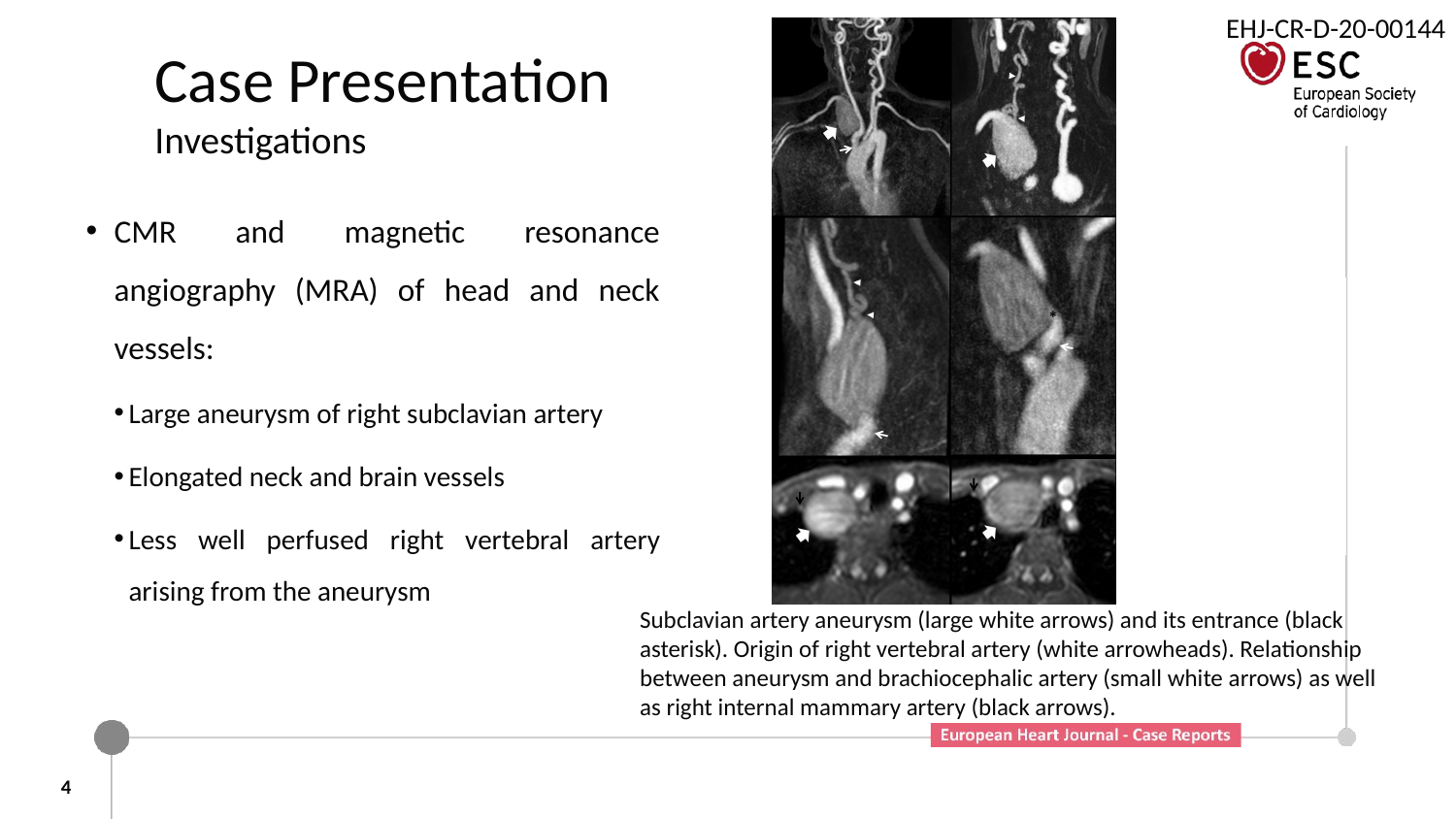

EHJ-CR-D-20-00144
# Case Presentation Investigations
CMR and magnetic resonance angiography (MRA) of head and neck vessels:
Large aneurysm of right subclavian artery
Elongated neck and brain vessels
Less well perfused right vertebral artery arising from the aneurysm
Subclavian artery aneurysm (large white arrows) and its entrance (black asterisk). Origin of right vertebral artery (white arrowheads). Relationship between aneurysm and brachiocephalic artery (small white arrows) as well as right internal mammary artery (black arrows).
4

## Slide 5
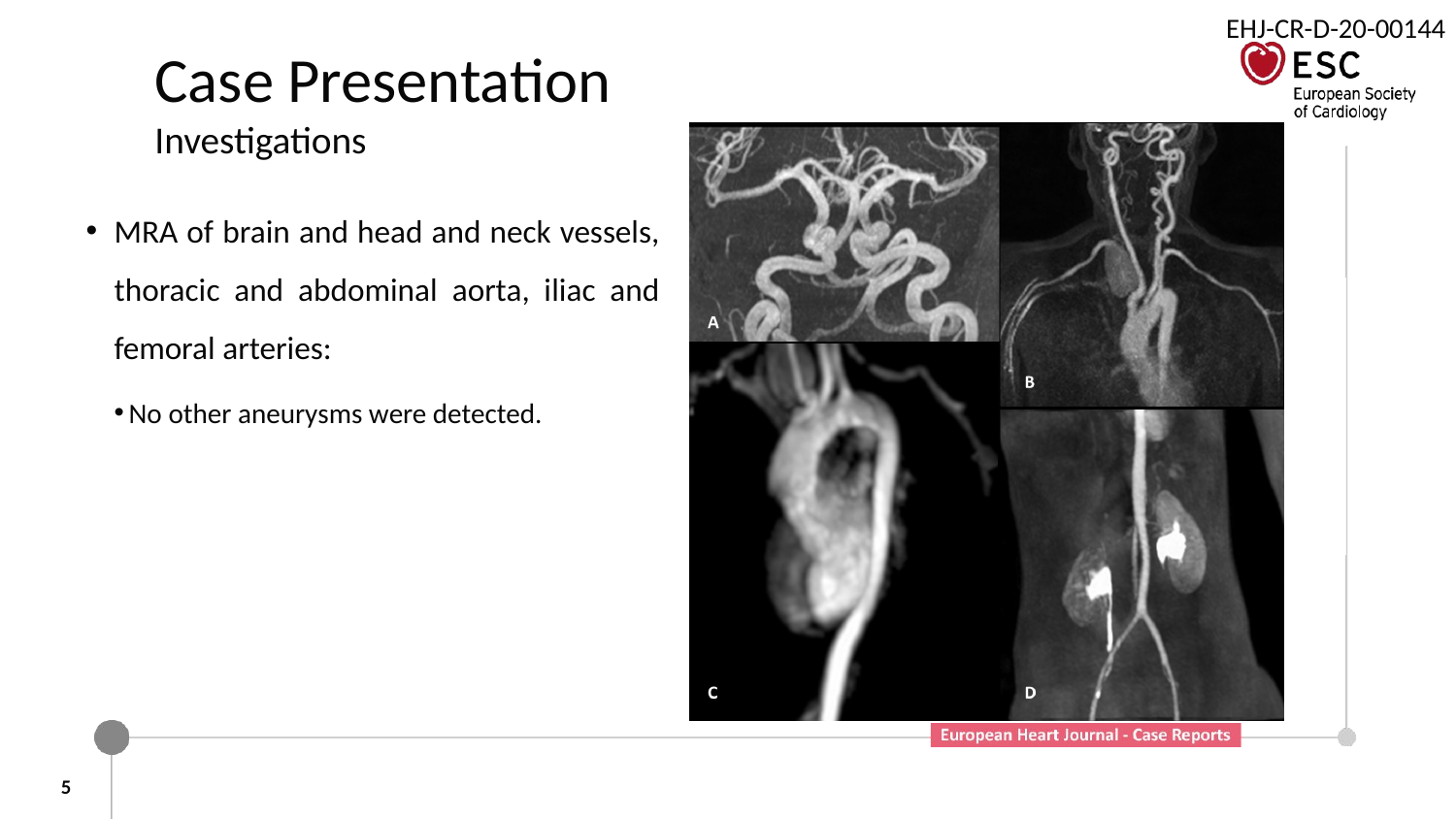

EHJ-CR-D-20-00144
# Case Presentation Investigations
MRA of brain and head and neck vessels, thoracic and abdominal aorta, iliac and femoral arteries:
No other aneurysms were detected.
5

## Slide 6
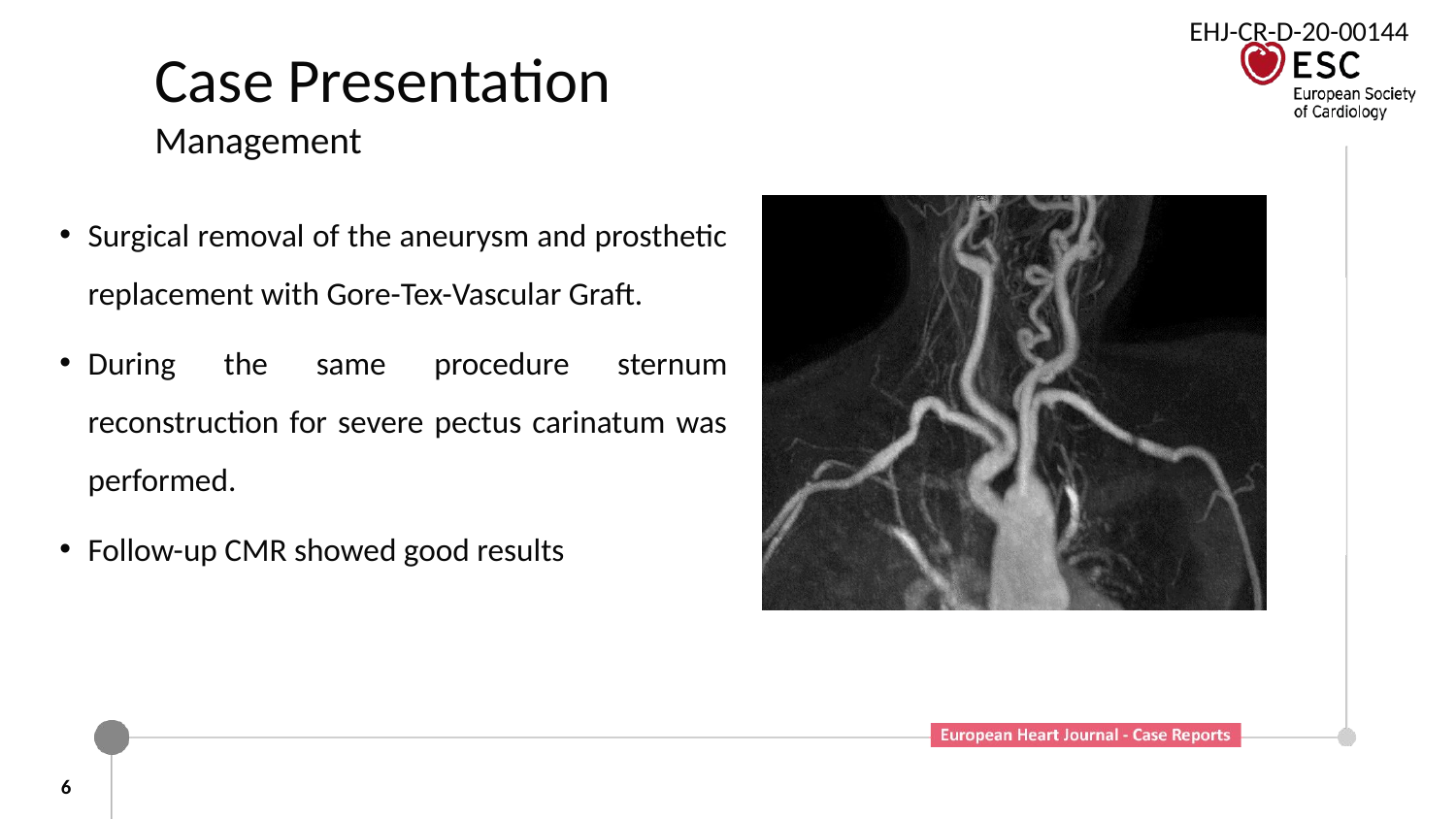

EHJ-CR-D-20-00144
# Case Presentation Management
Surgical removal of the aneurysm and prosthetic replacement with Gore-Tex-Vascular Graft.
During the same procedure sternum reconstruction for severe pectus carinatum was performed.
Follow-up CMR showed good results
6

## Slide 7
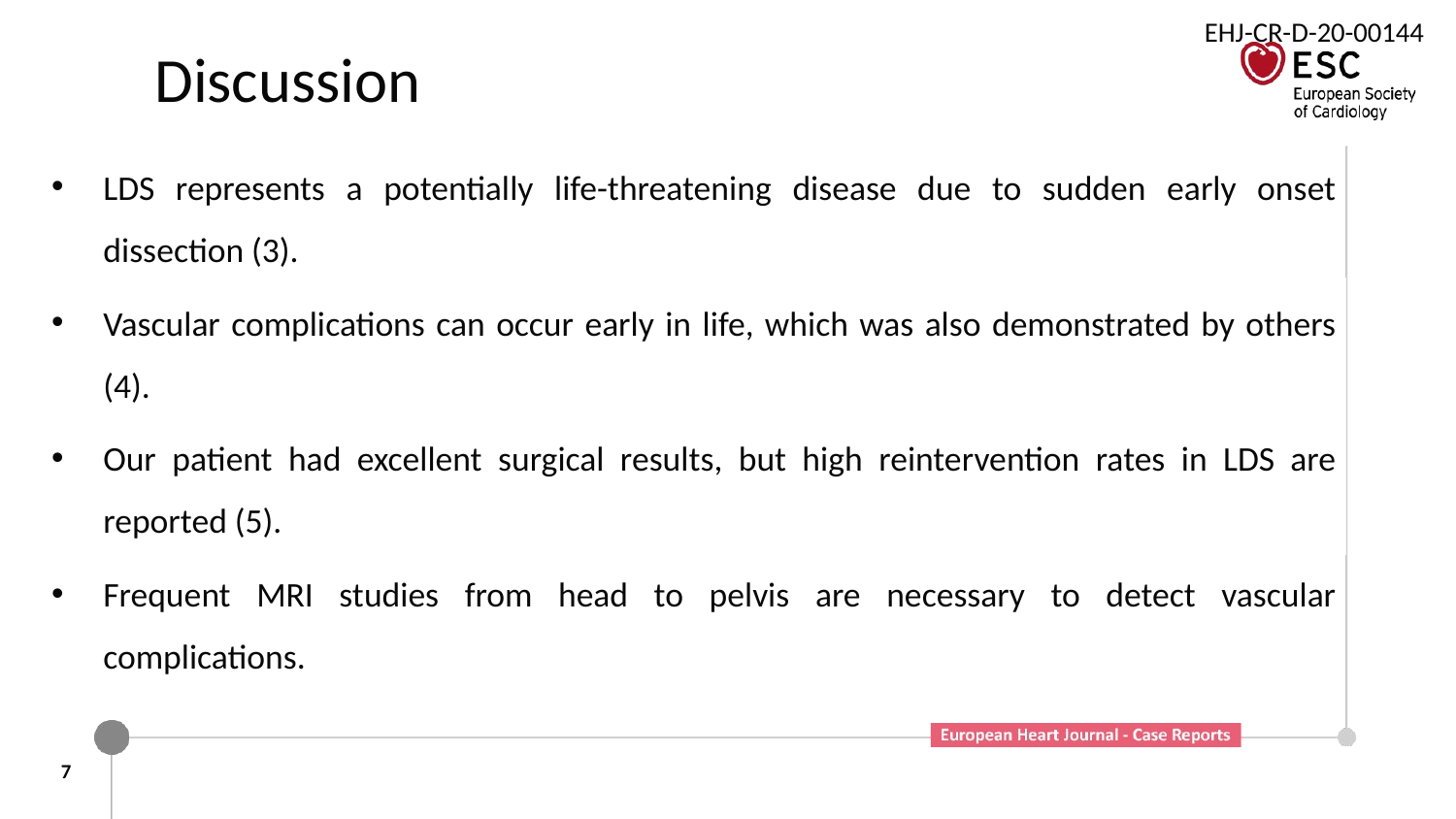

EHJ-CR-D-20-00144
# Discussion
LDS represents a potentially life-threatening disease due to sudden early onset dissection (3).
Vascular complications can occur early in life, which was also demonstrated by others (4).
Our patient had excellent surgical results, but high reintervention rates in LDS are reported (5).
Frequent MRI studies from head to pelvis are necessary to detect vascular complications.
7

## Slide 8
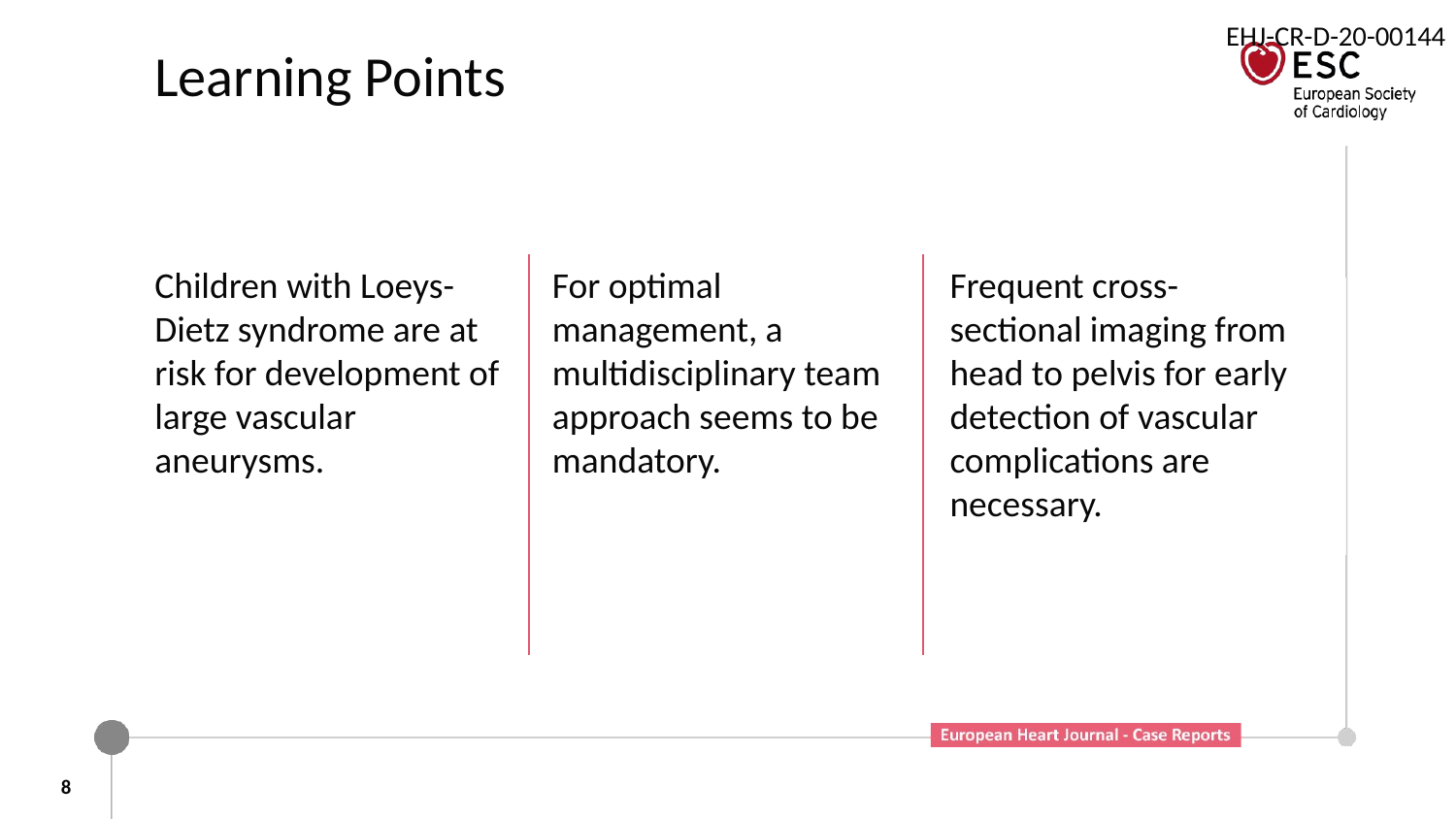

EHJ-CR-D-20-00144
# Learning Points
Children with Loeys-Dietz syndrome are at risk for development of large vascular aneurysms.
For optimal management, a multidisciplinary team approach seems to be mandatory.
Frequent cross-sectional imaging from head to pelvis for early detection of vascular complications are necessary.
8

## Slide 9
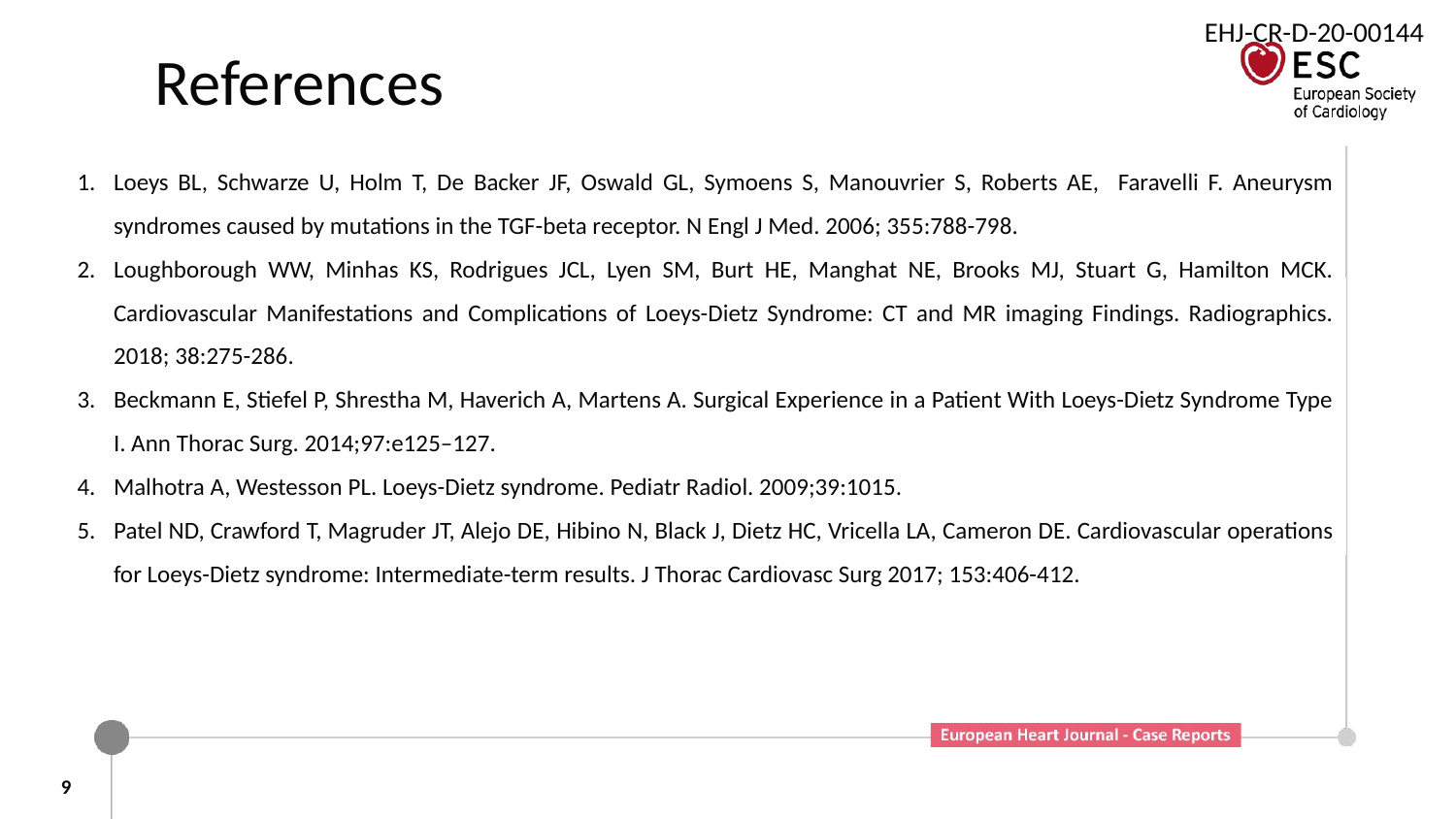

EHJ-CR-D-20-00144
# References
Loeys BL, Schwarze U, Holm T, De Backer JF, Oswald GL, Symoens S, Manouvrier S, Roberts AE, Faravelli F. Aneurysm syndromes caused by mutations in the TGF-beta receptor. N Engl J Med. 2006; 355:788-798.
Loughborough WW, Minhas KS, Rodrigues JCL, Lyen SM, Burt HE, Manghat NE, Brooks MJ, Stuart G, Hamilton MCK. Cardiovascular Manifestations and Complications of Loeys-Dietz Syndrome: CT and MR imaging Findings. Radiographics. 2018; 38:275-286.
Beckmann E, Stiefel P, Shrestha M, Haverich A, Martens A. Surgical Experience in a Patient With Loeys-Dietz Syndrome Type I. Ann Thorac Surg. 2014;97:e125–127.
Malhotra A, Westesson PL. Loeys-Dietz syndrome. Pediatr Radiol. 2009;39:1015.
Patel ND, Crawford T, Magruder JT, Alejo DE, Hibino N, Black J, Dietz HC, Vricella LA, Cameron DE. Cardiovascular operations for Loeys-Dietz syndrome: Intermediate-term results. J Thorac Cardiovasc Surg 2017; 153:406-412.
9
